# Invasive fungal disease in humans: are we aware of the real impact?

**DOI:** 10.1590/0074-02760200430

**Published:** 2020-10-09

**Authors:** Carolina Firacative

**Affiliations:** 1 Universidad del Rosario, School of Medicine and Health Sciences, Studies in Translational Microbiology and Emerging Diseases (MICROS) Research Group, Bogota, Colombia

**Keywords:** fungi, immunocompromised host, invasive fungal disease, risk factors

## Abstract

Despite the medical advances and interventions to improve the quality of life of those in intensive care, people with cancer or severely immunocompromised or other susceptible hosts, invasive fungal diseases (IFD) remain severe and underappreciated causes of illness and death worldwide. Therefore, IFD continue to be a public health threat and a major hindrance to the success of otherwise life-saving treatments and procedures. Globally, hundreds of thousands of people are affected every year with *Candida albicans*, *Aspergillus fumigatus*, *Cryptococcus neoformans*, *Pneumocystis jirovecii*, endemic dimorphic fungi and Mucormycetes, the most common fungal species causing invasive diseases in humans. These infections result in morbidity and mortality rates that are unacceptable and represent a considerable socioeconomic burden. Raising the general awareness of the significance and impact of IFD in human health, in both the hospital and the community, is hence critical to understand the scale of the problem and to raise interest to help fighting these devastating diseases.

It is estimated that the fungal kingdom has six million species widely distributed in the environment.^1^ Among these, several hundred species of both yeasts and molds can affect humans in several ways. The direct ingestion of poisonous mushrooms causes mycetismus, which can result in gastrointestinal tract disorders or even death. In cases of mycotoxicosis, humans can be also poisoned by the ingestion of food contaminated with fungal toxins (mycotoxins), which affect the liver, kidneys and other vital organs.^2^ Lastly, causing infection, fungi can be either primary or opportunistic pathogens of humans and the disease is called mycosis. Ranging from superficial infections of the hair, skin, nails or mucosal surfaces, which are usually benign, and other ailments including allergies, fungi can also cause invasive infections of internal organs that are progressive and lethal if not diagnosed and specifically treated.^3^ ^,^ ^4^ To cause invasive disease in humans, however, fungi must meet four criteria: (i) the ability to grow at or above mammalian body temperature; (ii) the ability to reach internal tissues by penetrating or evading host barriers; (iii) the ability to lyse tissues and absorb their components; and (iv) the ability to evade host immune defences.^5^ As warm-blooded animals with a highly sophisticated immune system, humans are naturally resistant to most invasive fungal diseases (IFD). As such, most cases of invasive disease occur in patients with an underlying serious illness or condition. Moreover, advances in medical care and life-saving treatments, which may lead to an impaired immune function, have increased the number of susceptible patients or people at-risk for fungal infections.^5^ Lastly, socioeconomic and geoecological characteristics, including the exposure to a high inoculum of fungi in their natural habitat, are also important elements that influence on the incidence and prevalence of fungal disease across the world.^6^

In this perspective article, the main risk factors of people to develop IFD are summarised, to aid recognising the impact of fungi on human health, an issue often underestimated and underappreciated. Considering that IFD are often disregarded during routine hospital care, it is very important to emphasise to microbiologists, diagnostic laboratory staff and clinicians, that not only the number but the diversity of patients at-risk for IFD are continuously rising, with mortality rates than remain unacceptably high.^3^ Additionally, most fungal infections are not reportable diseases, thus reliable information on local and global prevalence is not available. This review recalls the updated definition of IFD, collates recent epidemiological data and describes, in order of frequency, the most susceptible groups of people to acquire an IFD, namely those who undergo medical interventions, people with immunosuppression induced either by the treatment of an associated illnesses or by an underlying disease, people with bacterial or viral co-infections and immunocompetent individuals who acquire an IFD by environmental exposure.

Invasive fungal diseases

According to standard criteria, an invasive or systemic fungal disease is proven when tissue damage due to fungal elements is observed by histopathologic examination and/or when the aetiologic agent is isolated by culture from clinical sterile samples such as blood, tissue or cerebrospinal fluid.^7^ Infection can be initiated by invasion of fungal microbiota into the mucosa, by the inhalation of fungal spores from the environment or by direct inoculation, which lead to colonisation and dissemination. As clinical manifestations of IFD are not specific and the severity of the disease depends on the host’s defences and immune response, a high degree of suspicion is needed for the early diagnosis and optimal management of these infections.^8^

IFD are an emerging problem worldwide, are generally very difficult to cure and the associated mortality remains very high depending on the pathogen and patient population. Recent studies have estimated that globally, fungal infections kill more than 1.5 million people per year, which is similar to the mortality due to tuberculosis and about three-times more than malaria.^6^ However, the true burden of IFD is probably miscalculated because of the absence or availability of reliable, sensitive and universal diagnostic methods and because fungal infections are usually hindered by other diseases. Furthermore, when cases of fungal diseases are identified by a health provider, hospital or laboratory, they are not mandatory to be reported to public health departments; hence, most countries lack surveillance systems for IFD.

Among at-risk and immunocompromised patients, *Candida albicans*, *Aspergillus fumigatus*, *Cryptococcus neoformans*, *Pneumocystis jirovecii*, endemic dimorphic fungi and Mucormycetes remain the main fungal pathogens responsible for most cases of serious fungal diseases in the world (Tables I, II, Figure).^5^ ^,^ ^6^ ^,^ ^9^ ^,^ ^10^ While the ability of these fungi to cause disease depends on their virulence factors and pathogenic capacity, as well as on the interactions with the host and adaptation to different environments, the outcome of the infections is influenced by various others factors. The patients’ underlying medical condition, the time for diagnosis, the choice of therapy, especially when fighting strains or species that are resistant to antifungal drugs, the capacity to achieve prompt and effective source control, and the adverse effects of antifungal drugs alone or concomitantly administered with other drugs, are factors that significantly contribute to IFD prognosis.^5^ Thus, identifying susceptible patients or those who are clinically suspected of having a fungal infection, along with an early empiric therapy or prophylaxis, are of paramount importance to decrease mortality and morbidity associated to IFD. 

**TABLE I:** Major risk factors associated with the acquisition of an invasive fungal disease

Risk factor	Specific conditions	Most common pathogens
Medical intervention	Catheters, intravascular or intracranial devices	*Candida albicans*, *Candida* spp.
	Broad-spectrum antibiotics use	*C. albicans*, *Candida* spp.
	Neurosurgical procedures, contaminated devices and drug preparations	*Candida* spp., saprophyte fungi
Treatment-induced immunosuppression	Severe prolonged neutropenia	*Aspergillus fumigatus*, *Aspergillus* spp.
	Solid organ transplantation	*C. albicans*, *Candida* spp., *A. fumigatus*, *Cryptococcus neoformans*
	Haematopoietic stem cell transplantation	*A. fumigatus*, *C. albicans*, *Candida* spp., *Pneumocystis jirovecii*, Mucormycetes
	Biological agents	*Candida* spp., *Aspergillus* spp.*, Cryptococcus* spp., *P. jirovecii*, dimorphic fungi, Mucormycetes
Disease-induced immunosuppression	HIV infection	*P. jirovecii*, *C. neoformans*, *Histoplasma capsulatum, Talaromyces marneffei*
	Uncontrolled diabetes	*Rhizopus oryzae*, Mucormycetes
	Chronic obstructive pulmonary disease	*A. fumigatus*
Co-infection	Tuberculosis	*A. fumigatus, Aspergillus niger*, *H. capsulatum*, *C. neoformans*, *C. albicans*
	Cytomegalovirus	*P. jirovecii*, *Aspergillus* spp., non-*Aspergillus* molds
	SARS-CoV-2 infection	*A. fumigatus*
Environmental exposure	Trauma	*R. oryzae*, Mucormycetes

HIV: human immunodeficiency virus; SARS-CoV-2: severe acute respiratory syndrome coronavirus 2.

**TABLE II:** Estimated incidence and mortality rates of the most frequent invasive fungal diseases affecting humans

Mycosis	Main aetiologic agent	Cases per year	Mortality rate (%)
Invasive candidiasis	*Candida albicans*	~ 750,000^6^	~ 40^74^
Invasive aspergillosis	*Aspergillus fumigatus*	> 300,000^6^	30-70^23^ ^,^ ^24^
*Pneumocystis* pneumonia	*Pneumocystis jirovecii*	> 400,000^10^	10-60^38^
Cryptococcal meningitis	*Cryptococcus neoformans*	~ 225,000^44^	15 -50^74^
Disseminated histoplasmosis	*Histoplasma capsulatum*	~ 100,000^6^	10 -60^42^
Mucormycosis	*Rhizopus oryzae*	> 10,000^6^	35-100^49^

**Figure:**
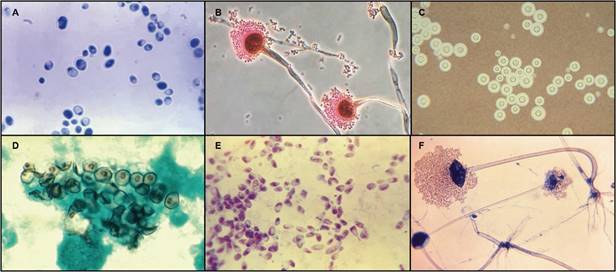
Photomicrographs of the most common fungal pathogens causing invasive diseases in humans. A: *Candida albicans* in its yeast-phase; B: filamentous conidiophores of *Aspergillus fumigatus*; C: encapsulated yeasts of *Cryptococcus neoformans*; D: *Pneumocystis jirovecii* cysts; E: *Histoplasma capsulatum* in its yeast phase; F: reproductive sporangia of *Rhizopus oryzae*. Images are not copyrighted and were obtained from the public domain of the Public Health Image Library (PHIL) from the Centers for Disease Control and Prevention (CDC) (phil.cdc.gov).^73^

Medical interventions

The placement of central venous catheters and intravascular or intracranial devices plus the use of broad-spectrum antibiotics, which are associated with medical interventions, are responsible for the vast majority of IFD acquired in hospital settings. Candidaemia, the most frequent life-threatening mycosis in the world, appears among the four most common causes of health care-associated bloodstream infections (BSI), with about 80% of cases occurring in the absence of evident immunosuppression but rather among patients with common iatrogenic and/or nosocomial conditions.^11^ ^,^ ^12^ Medical devices disrupt the normal skin barriers and provide direct access for the commensal yeasts of the genus *Candida* to the body’s interior. In addition, it has been long recognised that antibiotics suppress the growth of normal bacterial microbiota, which favours the proliferation of *Candida* species, thus leading to colonisation and an increased risk of disseminated candidiasis.^12^ ^,^ ^13^ *C. albicans* (A in Figure) and other *Candida* species also have the ability to form biofilms on indwelling venous and bladder catheters, heart valves, joint replacements and even on different tissues in the host. Biofilms are a physical barrier intrinsically resistant to the host immune system and other environmental perturbations.^14^ The formation of biofilms additionally leads to upregulation of antifungal-resistance mechanisms, as well as the development of complex regulatory processes that favour even higher levels of antifungal resistance, most notably to azoles and polyenes.^15^ Directly related with medical interventions, nosocomial candidaemia is associated with prolonged stays in hospital and intensive care units, parenteral nutrition, as well as with improved survival of premature infants.^11^ Despite the broad use of antifungal prophylaxis, mostly fluconazole, which has shown to reduce the incidence of invasive candidiasis in patients who are at highest risk, almost 750,000 cases of this mycosis are estimated to occur yearly in the world.^6^ ^,^ ^12^ Although *C. albicans* is the global leading agent of candidaemia and other forms of invasive candidiasis, accounting for about half the amount of cases, the frequency of non-*albicans* species, mainly *C. glabrata, C. parapsilosis*, *C. tropicalis* and *C. krusei* is increasing, which is of concern when selecting empiric antifungal therapy, as non-*albicans* species have reduced susceptibility or are intrinsically resistance to azoles.^11^

Much less frequently, in both immunocompetent and immunocompromised individuals, fungi, including *Candida* spp., can be directly inoculated through neurosurgical procedures and contaminated devices or drug preparations. An unprecedented outbreak of fungal meningitis, for instance, was associated with parenteral injections of corticosteroids contaminated with *A. fumigatus* and *Exserohilum rostratum*, both environmental molds.^16^ ^,^ ^17^ Another saprophyte mold, *Sarocladium kiliense*, was isolated from several fungaemia cases associated with the administration of contaminated antinausea medication among oncology patients.^18^ ^,^ ^19^

Treatment-induced immunosuppression

Haematological malignancies, mainly acute leukaemia, account for most cases of IFD among patients immunosuppressed in the classical sense. Prolonged severe neutropenia, resulting from intense chemotherapy regimens and defined as more than 10 days with a peripheral blood absolute neutrophil count less than 500/µL, is still the single major risk factor for invasive infection caused by the environmental mold *A. fumigatus* (B in Figure) (> 80% of cases) and other species of *Aspergillus*.^9^ ^,^ ^20^ Neutrophils are known to be the primary host defence mechanism against *Aspergillus* infection, which commonly starts by the inhalation into and establishment of fungal spores in the lower respiratory tract. When mucosal barriers have been injured, neutrophils function in the innate immune response through the killing of conidia and hyphae by both oxidative and nonoxidative mechanisms.^21^ ^,^ ^22^ In neutropenic patients, therefore, neutrophil dysfunction represents an impairment to efficiently recruit and kill *Aspergillus* infectious elements. Worldwide, it is estimated that invasive aspergillosis affects annually more than 300,000 patients from almost 10 million at risk.^6^ Despite the availability of highly active antifungal treatment and the implementation of prevention strategies such as the introduction of mold-active prophylaxis and the reduction of exposure to *Aspergillus* spores, invasive aspergillosis in haemato-oncological patients is still associated with extremely high mortality rates (60-70%).^23^ ^,^ ^24^

Similarly, thousands of solid organ transplant (SOT) recipients in the world are at risk of IFD as a result of organ damage, neutropenia, administration of immunosuppressive drugs and surgical factors such as prolonged operation time, increased technical complexity and bleeding complications. In these patients, *Candida* spp.*, A. fumigatus* and *C. neoformans* (C in Figure) are reported as the most common fungal pathogens causing invasive disease, although the incidence of other molds, such as *Fusarium* spp. and dimorphic fungi, is increasing.^25^ ^,^ ^26^ In SOT recipients, however, the risk for and distribution of IFD depend on the organ transplanted. It has been reported that kidney transplant recipients had the lowest overall risk of IFD and lung transplant recipients are more commonly affected by *Aspergillus* spp.^26^ ^,^ ^27^ Survival of transplanted patients who developed an IFD also differs depending on the aetiological agent causing the infection, with candidaemia and other forms of invasive candidiasis generally accounting for higher number of deaths.^25^ ^,^ ^26^ While invasive candidiasis is generally an early complication of SOT, aspergillosis, cryptococcosis and other fungal infections tend to present later after transplantation. In addition, time to onset of IFD may also differ based on antifungal prophylaxis, site of infection or transplant type.^28^

Cytopaenia and administration of therapies to prevent and treat graft-*versus*-host disease (GvHD), which leads to an impaired cell immunity, are major risk factors of haematopoietic stem cell transplant (HSCT) recipients for acquiring IFD.^29^ ^,^ ^30^ Due to induced neutropenia and lack of adequate immune reconstitution, invasive aspergillosis has been noted to be the most commonly observed IFD in HSCT recipients, followed by invasive candidiasis, *Pneumocystis* pneumonia, mucormycosis (caused by Mucormycetes) and infections caused by other molds such as *Fusarium* and *Scedosporium* species.^31^ ^,^ ^32^ Gastrointestinal tract mucositis, defined as the disruption of the gastrointestinal tract mucosa, together with the presence of venous catheters in patients who require transfusion support, allow for gut and skin microbiota, including *Candida* spp., to invade and cause BSI.^30^ ^,^ ^32^ As in SOT recipients, IFD attributable mortality rates differ depending on the aetiological agent causing the infection, with non-*Aspergillus* mycosis and mucormycosis being responsible for most fatal cases in stem cell transplanted patients.^31^ ^,^ ^33^ Donor type, GvHD grade, conditioning regimens, supportive care strategies and therapy timing are factors that also influence the risk for IFD in HSCT recipients.^30^ Clearly, invasive mycoses hamper the success of the transplantation and continue to be major threats to patients undergoing SOT and HSCT.

Biologic agents, such as antagonists of the tumour necrosis factor (TNF)-a, which are used as therapeutic approaches for autoimmune conditions including rheumatoid arthritis, juvenile idiopathic arthritis, psoriatic arthritis, ankylosing spondylitis, plaque psoriasis and inflammatory bowel diseases directly or indirectly impact the immune response against fungal pathogens, mainly by affecting inflammatory processes, thus increasing the risk of IFD.^34^ ^,^ ^35^ The immune response against fungal pathogens can be impaired by the blockade of TNF-a, which leads to reduced cytokine production, impairment of monocyte recruitment, prevention of granuloma formation and apoptotic and nonapoptotic death of cells expressing TNF receptors.^34^ Although baseline predisposition of patients for fungal infections due to the autoimmune disorders, concomitant immunosuppressive therapies and geographical location need to be considered, *Aspergillus*, *Candida* and *Cryptococcus* infections are generally associated with the use TNF-a inhibitors. A study reported that among people receiving these biologic agents, candidiasis was the most frequent mycosis followed by histoplasmosis, aspergillosis, cryptococcosis and coccidioidomycosis, with incidences that ranged between 5.1 to 13.3 cases per 100,000 persons treated, over a 4.75-year period.^36^ Other studies, however, reported *P. jirovecii*, a unculturable yeast-like fungus, as the most common nonviral opportunistic infection, with 16 cases among 33,324 users of TNF-a inhibitors.^37^ More rarely, agents of mucormycosis, other dimorphic fungi and non-*Aspergillus* molds have been reported in patients on active biologic therapy.^34^

Disease-induced immunosuppression

Defects in cell-mediated immunity, mainly a decrease in the number and function of CD4^+^ lymphocytes, which occurs in people infected with the human immunodeficiency virus (HIV), is the major risk factor for *Pneumocystis* pneumonia. Among people living with HIV, *P. jirovecii* (D in Figure) persists as the most common cause of serious and often fatal respiratory infection, that continues to be a life-threatening defining illness in patients with acquired immunodeficiency syndrome (AIDS).^38^ Yearly, more than 400,000 cases of *Pneumocystis* pneumonia are estimated to occur worldwide, with mortality rates ranging from 10-60% or higher, depending on the patient population, comorbidities and time for diagnosis.^10^ ^,^ ^38^ Unlike other fungal species affecting humans, evidence suggests that *P. jirovecii* colonises the respiratory tract of asymptomatic individuals and that person-to-person transmission is the most likely mode of infection, which is of clinical significance as colonised individuals may be at risk of development pneumonia or may transmit *Pneumocystis* to others.^38^ ^,^ ^39^

Low CD4^+^ counts is also the most important risk factor to acquire infection by *C. neoformans*, an environmental encapsulated yeast.^40^ As *C. neoformans* has a special predilection for the central nervous system (CNS), the main presentation of cryptococcosis is meningoencephalitis, which if left untreated, is deadly or leads to long-term neurological sequelae.^5^ ^,^ ^41^ Although the introduction of antiretroviral treatments (ART) for people living with HIV has reduced the global incidence of cryptococcosis, the limited access to healthcare and the increasing number of HIV-positive people contribute to the high prevalence of cryptococcal infection in low- and middle-income countries.^42^ In patients with a CD4^+^ count less than 100 cells/μL who are not receiving effective ART, screening for asymptomatic cryptococcal antigenaemia combined with pre-emptive antifungal therapy with fluconazole has proven to be an effective strategy to prevent symptomatic meningitis whilst reducing the number of deaths.^43^ Nevertheless, worldwide there are circa 225,000 estimated new cases of cryptococcal meningitis per year, causing over 180,000 deaths, which accounts for about 15% of AIDS-associated deaths.^44^ In addition, concomitant treatment with antiretroviral drugs in patients with AIDS-related cryptococcosis can cause exaggerated inflammatory response as a result of immune reconstitution inflammatory syndrome, which contributes to even higher mortality rates.^40^ ^,^ ^45^

Histoplasmosis and talaromycosis, caused by the thermal dimorphic fungi *Histoplasma capsulatum* (E in Figure) and *Talaromyces* (formerly *Penicillium*) *marneffei*, respectively, are also major opportunistic infections affecting patients with HIV/AIDS, which present mostly as disseminated disease.^42^ Disseminated histoplasmosis, reported worldwide with about 100,000 cases occurring every year, has most endemic cases in the Americas, where the incidence has been estimated to reach 25% of patients with HIV infection, being an AIDS-defining illness in up to 75% of patients and with mortality rates of almost 60%.^6^ ^,^ ^42^ ^,^ ^46^ In Latin America, it is estimated that, among people living with HIV, the number of deaths caused by histoplasmosis may be higher than those caused by tuberculosis. In fact, histoplasmosis, which is considered neglected in this region, is often misdiagnosed as tuberculosis^47^ Talaromycosis, restricted to southeast Asia, southern China and northeastern India, remains one of the major causes of HIV-associated mortality in these endemic areas, especially in people with undiagnosed and untreated HIV infection.^42^ ^,^ ^46^ ^,^ ^48^

Uncontrolled diabetes *mellitus*, particularly in patients having ketoacidosis, is the major predisposing factor for developing mucormycosis, which is the third most common angioinvasive fungal infection following candidiasis and aspergillosis.^49^ ^,^ ^50^ Mucormycetes species are vasotropic and cause tissue infarctions.^49^ Among them, *Rhizopus oryzae* (F in Figure) is the most frequently recovered species in patients with mucormycosis (∼ 70%), followed by *Mucor* spp., *Rhizomucor* spp., *Syncephalastrum* spp., *Cunninghamella bertholletiae*, *Apophysomyces elegans*, *Lichtheimia* spp., and *Saksenaea* spp., among other numerous species.^51^ After inhalation of spores from environmental sources, immunocompromised hosts can develop upper and lower respiratory disease, which if left untreated, can subsequently spread to the CNS causing rhinocerebral mucormycosis.^52^ In people with diabetes, monocytes and macrophages are dysfunctional and fail to phagocyte and kill the Mucormycetes spores, allowing their germination and subsequent proliferation as hyphal elements, which leads to rapid invasion of deeper tissues and dissemination.^53^ Despite several simultaneous approaches used for treatment, such as aggressive debridement or amputation, antifungal therapy and medical management or correction of the underlying condition, mucormycosis associated mortality rates continue to be very high, reaching almost 100% among patients with disseminated disease.^49^ ^,^ ^52^

Chronic obstructive pulmonary disease (COPD) represents an important, nonclassical underlying condition for the increasing number of acute invasive pulmonary aspergillosis, with a very high mortality rate. Associated with the pulmonary disease, structural modifications in the lung architecture that favour lung parenchyma invasion and necrosis due to *Aspergillus* spp., in addition to prolonged use of immunosuppressive treatment, such as corticosteroid therapy, might increase the susceptibility of COPD patients to acquire an IFD.^54^ Furthermore, recurrent hospitalisation, invasive procedures, broad-spectrum antibiotic use and other factors such as tobacco smoke, alcoholism, diabetes *mellitus* and/or malnutrition could contribute to promote invasive aspergillosis in COPD patients.^54^ ^,^ ^55^

Bacterial and viral co-infections

Fungal pulmonary infections can be acquired primarily or secondarily during tuberculosis infection. Co-morbidities such as immunodeficiency, mainly due to HIV, chronic illnesses and malignancy, together with inherent factors of the community like poverty and malnutrition, worsen the co-infection and the prognosis of patients.^56^ In 2018, it was estimated that globally 10 million people contracted tuberculosis, causing 1.2 million deaths among HIV-negative people and 251,000 deaths associated with HIV infection.^57^ As fungal pulmonary disease does not show specific radiological and clinical manifestations, compared to those of pulmonary tuberculosis, misdiagnosis and late or no-antifungal therapy cause high morbidity and mortality rates in co-infected patients. With an incidence that can reach 50% of cases, depending on the geographical distribution, different fungal pathogens coexist with pulmonary tuberculosis, amplifying the severity of the disease, especially when dissemination occurs. *A. fumigatus*, *Aspergillus niger*, *H. capsulatum*, *C. neoformans* and *C. albicans* are often responsible for causing severe secondary infections in tuberculosis patients.^10^ ^,^ ^56^ ^,^ ^58^ ^,^ ^59^

Reactivation of latent cytomegalovirus (CMV) can lead to the development of concomitant or sequential fungal infections in immunosuppressed patients. Both solid organ and autologous stem cell transplant recipients, with a history of CMV infection, have higher risk for developing IFD.^60^ ^,^ ^61^ In HSCT patients, *Pneumocystis* pneumonia and invasive mold infections, including invasive aspergillosis, represent the most commonly encountered IFD during the late post-engraftment period.^32^ ^,^ ^61^ Untreated CMV viremia, which occurs primarily in patients who were seropositive before transplantation and who develop GvHD, is followed by pneumonia that may be accompanied by superinfection with fungal pathogens, leading to substantial morbidity and mortality.^30^

A dysfunctional immune response, which can cause severe alveolar lung damage and even systemic pathology, has been reported to occur rarely in people with coronavirus disease 2019 (COVID-19), a viral infection caused by the severe acute respiratory syndrome coronavirus 2 (SARS-CoV-2), first reported in Wuhan, China, at the end of 2019.^62^ ^,^ ^63^ While unusual, this harmful interaction of SARS-CoV-2 with the immune system of patients with severe COVID-19 pneumonia seems to increase the susceptibility of these patients to acquire secondary infections, including certain mycoses.^62^ Although the incidence of IFD associated with COVID-19 is still unknown, *Aspergillus* infections have been, so far, the most commonly reported fungal co-infections in COVID-19 patients.^64^ Invasive aspergillosis, however, has been associated with admission to critical care because of COVID-19 pneumonia and severe acute respiratory syndrome.^65^ In addition, most co-infected patients had previous history of underlying immunocompromised status or pre-existing comorbidities, mainly hypertension, diabetes, obesity and haematological malignancies.^65^ ^,^ ^66^ Much less frequently, candidiasis has been also reported as co-infection in hospitalised patients with COVID-19.^67^ ^,^ ^68^ ^,^ ^69^ However, the contribution of the underlying disease and COVID-19, as attributable risk factors to acquire fungal infections requires careful analysis and to date remains to be determined.^64^

Environmental exposure

Environmental factors play a unique role in the epidemiology of invasive mold infections. Although infrequent, these opportunistic infections are serious complications of both traumatic and post-traumatic injury.^70^ Patients can be directly inoculated during tissue injury; alternatively, fungi can infect the tissue after skin trauma, burns or surgery. In all patients, however, cutaneous manifestations are followed by angioinvasion, which results in vessel thrombosis and tissue necrosis.^53^ When the patient has any kind of immunosuppression besides the traumatic injury, tissue infarction and necrosis occur usually shortly after the inoculation event.^70^ Globally, mucormycosis is the most common fatal complication that can occur after trauma, because of the quick dissemination of spores and deep extension to bone, tendon, or muscle, after skin disruption.^51^ Upon inoculation, *R. oryzae* obtains iron from the host, which is abundant in patients with strong haemorrhage and/or receiving blood transfusions.^52^ Mucormycetes have been reported causing outbreaks of traumatic implantation of contaminated soil or water after natural disasters, including tsunamis and tornadoes. Motor vehicle collisions and blasts from war-related injuries are also common forms to get severe trauma among the population.^71^ After dissemination occurs, even young, previously healthy patients are susceptible to mucormycosis and even with aggressive approaches such as disfiguring debridement or amputation, case-fatality rate can reach 100%.^71^ ^,^ ^72^

In conclusion, concomitant with the increased prevalence of at-risk and immunocompromised people, rates and spectrum of invasive fungal infections are rising and becoming more frequent worldwide. Certainly, opportunities are increasing for opportunistic fungi. Extended survival of patients related to improvements in supportive and intensive care, prolonged treatment of bacterial infections, high-dose chemotherapeutic regimens to treat cancers, global practice of solid organ and stem cell transplantation procedures, increased use of immunosuppressive and biologic therapies, the advent of AIDS, and even natural disasters, among other factors, significantly contribute to the impact of fungal pathogens on human health. Although advances in medical technology, improved diagnostics, earlier administration of antifungal treatments and more effective preemptive therapy have helped reducing morbidity and mortality for most IFD, fungal opportunistic infections persist severe, often underdiagnosed, life-threatening and have a significant economic burden. Therefore, as fungi are ubiquitous in the environment and they are increasingly infecting vulnerable populations worldwide, especially those living in resource-limited settings, there is an urgent need to increase clinical awareness, in order to develop early intervention strategies. Identifying major risk factors or conditions is of utmost importance to prevent IFD and meet the need of decreasing the still staggering high mortality-associated rates. Moreover, even though IFD are among the deadliest communicable diseases worldwide, the vast majority of these mycoses are not reportable to public health authorities in most countries, which represents another challenge and makes IFD less likely to be documented. Whilst waiting for mandatory case reporting, comprehensive eco-epidemiological studies together with voluntary reports from clinicians and laboratories are needed, aimed to improve disease surveillance and to achieve a better understanding of the public health implications of IFD around the world. Obtaining a true picture of the global burden of invasive mycoses, including the patients’ associated conditions and risk factors, is key to making decisions and providing inputs into health-care services, programmes, responses and policies, which will ultimately be addressed to improving people’s health and wellbeing.
